# Temperature-Dependent Gene Expression in *Yersinia ruckeri*: Tracking Specific Genes by Bioluminescence During *in Vivo* Colonization

**DOI:** 10.3389/fmicb.2018.01098

**Published:** 2018-05-25

**Authors:** Jessica Mendez, Desirée Cascales, Ana I. Garcia-Torrico, Jose A. Guijarro

**Affiliations:** Área de Microbiología, Departamento de Biología Funcional, Facultad de Medicina, Instituto de Biotecnología de Asturias, Universidad de Oviedo, Oviedo, Spain

**Keywords:** *Yersinia ruckeri*, temperature regulated genes, bioluminescence, *acrR*, osmY

## Abstract

*Yersinia ruckeri* is a bacterium causing fish infection processes at temperatures below the optimum for growth. A derivative *Tn5* transposon was used to construct a library of *Y. ruckeri* mutants with transcriptional fusions between the interrupted genes and the promoterless *luxCDABE* and *lacZY* operons. *In vitro* analysis of β-galactosidase activity allowed the identification of 168 clones having higher expression at 18°C than at 28°C. Among the interrupted genes a SAM-dependent methyltransferase, a diguanylated cyclase, three genes involved in legionaminic acid synthesis and three transcriptional regulators were defined. In order to determine, via bioluminescence emission, the *in vivo* expression of some of these genes, two of the selected mutants were studied. In one of them, the *acrR* gene coding a repressor involved in regulation of the AcrAB-TolC expulsion pump was interrupted. This mutant was found to be highly resistant to compounds such as chloramphenicol, tetracycline, and ciprofloxacin. Although *acrR* mutation was not related to virulence in *Y. ruckeri,* this mutant was useful to analyze *acrR* expression in fish tissues *in vivo*. The other gene studied was *osmY* which is activated under osmotic stress and is involved in virulence. In this case, complemented mutant was used for experiments with fish. *In vivo* analysis of bioluminescence emission by these two strains showed higher values for *acrR* in gut, liver and adipose tissue, whereas *osmY* showed higher luminescence in gut and, at the end of the infection process, in muscle tissue. Similar results were obtained in *ex vivo* assays using rainbow trout tissues. The results indicated that this kind of approach was useful for the identification of genes related to virulence in *Y. ruckeri* and also for the *in vivo* and *in vitro* studies of each of the selected genes.

## Introduction

*Yersinia ruckeri* is the etiological agent of “Enteric red mouth disease” of salmonids which causes important economic losses in the aquaculture industry. The bacterium is distributed worldwide and four serotypes were defined (Romalde et al., 1993). The most virulent is serotype O1 which has two biotypes 1 and 2 (Davies and Frerichs, 1989). Most outbreaks are caused by biotype 1, but in recent years biotype 2 has been associated with outbreaks in different parts of the world (Arias et al., 2007; Calvez et al., 2014).

A key environmental stress factor in outbreaks of most bacterial fish diseases in fish farms is water temperature. In some cases, outbreaks occur when water temperature drops to a certain value, as in the case of “Cold water vibriosis” (Enger et al., 1991) and “Cold water disease” (Cipriano and Holt, 2005). In other diseases, such as “lactococcosis,” outbreaks are related to an increase in water temperature (Vendrell et al., 2006). Interestingly, a remarkable number of bacterial diseases in aquaculture, particularly those of freshwater, occurred at temperatures below the optimum for growth of the infecting bacteria (Guijarro et al., 2015). Bacterial genes activated under these conditions were identified in fish pathogens. Indeed, in *Flavobacterium psychrophilum*, Hesami et al. (2011) defined a set of genes that were up-regulated at 8°C versus 20°C by using suppression subtractive hybridization. In the same way, in *Lactococcus garvieae* several genes linked to virulence were up-regulated at 18°C versus 37°C (Aguado-Urda et al., 2013). In *Aeromonas hydrophila* a MALDI-TOF analysis of the extracellular products showed that a serine-metalloprotease, S-layer, flagellins and proteins related to the type III secretion system were up-regulated at 25°C versus 37°C (Yu et al., 2007). The two-component system PhoP-PhoQ of *Edwardsiella tarda* responds to changes in environmental temperature by activating type III and type VI secretion systems, both associated with virulence of the bacterium (Srinivasa Rao et al., 2003, 2004; Zheng et al., 2005; Wang et al., 2009; Chakraborty et al., 2010). In spite of these studies, little is known about the temperature-regulated virulence factors in fish-associated bacterial pathogens and even less about the systems involved in their regulation.

*Y. ruckeri* has an optimal growth temperature of 28°C but outbreaks of disease occur at temperatures around 18°C. Virulence of *Y. ruckeri* is multifactorial and different genes were described as being involved in pathogenesis (Fernández et al., 2007a; Tobback et al., 2007; Kumar et al., 2015). Expression of some of these genes was higher at 18°C than at 28°C (Fernández et al., 2004). Thus, the expression of the *traH-N* operon encoding a putative type IV secretion system (Méndez et al., 2009), YhlA hemolysin (Fernández et al., 2007b), Yrp1protease (Méndez and Guijarro, 2013) and ruckerbactin, a catecholate siderophore iron acquisition system were up-regulated at 18°C versus 28°C (Fernández et al., 2004). Therefore, the temperature-dependent modulation of virulence genes in *Y. ruckeri* tends to optimize the expression of these in conditions mimicking those encountered in the host.

As opposed to human pathogenic *Yersinia* species in which virulence factors are induced at temperatures near the optimal for bacterial growth, there is no study related to how *Y. ruckeri* regulates virulence gene expression at temperatures below the optimum for growth. The results of previous studies carried out in *Y. ruckeri* (Fernández et al., 2004; Méndez and Guijarro, 2013), showed the need for the present investigation, whose goal was the selection and identification of *Y. ruckeri* genes expressed preferentially at 18°C versus 28°C due to their potential role as virulence factors. Using transcriptional fusions between *Y. ruckeri* promoters and *lux-lac* operons inserted in the *Tn5* transposon, 168 clones having higher β-galactosidase activity at 18°C than at 28°C in an EMB medium were selected. Two of them, carrying *Tn5 lux-lac* insertions in the *acrR* and *osmY* genes, a repressor of the AcrAB-TolC system and a gene induced under osmotic shock, respectively, were further characterized both *in vitro* and *in vivo*. This study presents a useful, practical approach for the identification and further analysis of genes involved in virulence and for the determination of their expression both *in vitro* and *in vivo*.

## Materials and Methods

### Bacterial Strains, Plasmids, and Culture Conditions

*E. coli* strains (**Table 1**) were routinely grown in 2x TY (16 g/L tryptone, 10 g/L yeast extract, 5 g/L NaCl) broth and 2% agar, and *Y. ruckeri* strains (**Table 1**) in nutrient broth (NB) or nutrient 1.5% agar (NA) from VWR International and Tryptic Soy Broth (TSB) and Tryptic Soy 1.5% agar (TSA) from Merck. Liquid cultures were incubated at 37°C for *E. coli* and at 18°C and 28°C for *Y. ruckeri* in orbital shakers at 250 rpm. Growth was monitored by determining the OD_600_. In order to detect changes in the β-galactosidase activity, EMB from Merck was used. This is a differential microbiological medium useful to distinguish between organisms that ferment lactose (β-galactosidase positive) and those that do not (β-galactosidase negative). The lactose fermenters would produce dark colonies whereas the non-fermenters would form translucent or pink ones. When required, the following compounds were added to the media: 10 μg/mL erythromycin, 0.1 μg/mL cefotaxime, 50 μg/mL kanamycin, 50 μg/mL streptomycin, or 100 μg/mL ampicillin, all from Sigma-Aldrich Co.

**Table 1:** Characteristics of the strains and plasmids used in this work.

Strain or plasmid	Relevant properties	Source or reference
*Strains*		
*E. coli*		
S17-1λpir	λ(pir) hsdR pro thi RP4-2 Tc::mu Km::Tn7	Simon et al., 1983
DH5 αλpir	F‘/endA1 hsdR17 (rk-mk+) supE44 thi-1 recA1 gyrA (Nal^R^) λ (pir)	Woodcock et al., 1989
*Y. ruckeri*		
150	Strain of serotype O1, biotype 1 isolated from an outbreak in trout.	J. L. Larsen, (Denmark)
150CTX	Strain 150 cultured in presence of cefotaxime	This work
150 *acrR^-^*	*acrR*::mini-Tn5 *luxlac* Km2, Km^r^	This work
150 *acrR^+^*	*acrR^-^* con pGBM5::*acrR*	This work
150 *osmY^-^*	*osmY*::mini-Tn5 *luxlac* Km2, Km^r^	This work
150 *osmY^+^*	*osmY^-^* harboring pGBM5::*osmY*	This work
150 *osmY^++^*	*osmY^-^* harboring pGBM5::*osmY*-*ytjA*	This work
150 β1T.B2	Mini Tn5-luxlac mutant cultured in presence of cefotaxime	This work
Plasmids		
pCS26Pac	Km^r^, *luxCDABE*	Bjarnason et al., 2003
pGBM5	Spc^r^/Sm^r^, promoter lac	Manen et al., 1997
pIVET8	Ap^r^, oriR6K, mob+, cat-lacZY promotorless	Mahan et al., 1995
pUC19	Ap^r^, cloning vector	Pharmacia
pUT mini-Tn5 Km2	Ap^r^, oriR6K, mobRP4, tnp, mini-Tn5 Km2 (Km^r^)	de Lorenzo et al., 1990
pUT mini-Tn5 *lac* Km2	pUT mini-Tn5 Km2 harboring *trpAlacZY* genes without promoter or transcription termination sequences	This work
pUT mini-Tn5 *luxlac* Km2	pUT mini-Tn5 Km2, harboring *luxABCDE* and *trpAlacZY* tandem genes without promoter	This work

### Construction of Mini-*Tn5 lux-lac Km2* Transposon

Construction of pUT mini-*Tn5 lux-lac Km2* (**Table 1**) was initiated by utilizing the pUT mini-*Tn5 Km2* vector (de Lorenzo et al., 1990). First, a 6 Kb SphI DNA fragment harboring the *lacZY* operon lacking the promoter and terminator was removed by enzymatic digestion from the pIVET8 plasmid (Mahan et al., 1995) (**Table 1**) and ligated to the pUT vector, previously digested with the same enzyme and dephosphorylated. The ligation mixture was introduced in *E. coli* S17-1*λpir* by electroporation. Transformed cells were selected on 2x TY media supplemented with ampicillin. The correct orientation of the *lacZY* operon was defined by DNA sequencing using the Lacsec primer (Supplementary Table S1). Once the pUT mini-*Tn5 lac Km2* vector was obtained, a 5.8 Kb NotI DNA fragment containing the *luxCDABE* operon was removed from the pCS26PAC vector (Bjarnason et al., 2003) and ligated into the NotI restriction size located at the 5′end of the *lacZY* operon in the pUT mini-*Tn5 lac Km2* vector. The ligation mixture was introduced by electroporation in *E. coli* S17-1*λpir* and clones were selected on 2x TY media containing ampicillin. Random clones were used for plasmid purification and further analysis by PCR and DNA sequencing in order to select those carrying the *lux* operon in the 5′-3′orientation with respect to *lacZY* and kanamycin-resistant genes. The obtained plasmid was named pUT mini-*Tn5 lux-lac Km2* (**Table 1**).

In brief, pUT mini-*Tn5 lux-lac Km2* plasmid was transferred from *E. coli* S17-1*λpir* to *Y. ruckeri* 150 by conjugation. To do that, 500 μl of donor and 4 mL of recipient strains in exponential growth phase (OD_600_, 0.6) were washed twice by centrifugation and resuspended in 10 mL of MilliQ water. Then, the suspension was filtered through a 0.45 μm pore membrane that was transferred onto a 2x TY medium and incubated for 4 h at 28°C. After incubation, the bacteria were resuspended in 2 mL of NB medium and aliquots of 50 μL were spread onto NA medium containing kanamycin (resistance to which was conferred by the transposon) and erythromycin or cefotaxime, antibiotics to which *Y. ruckeri* 150 is intrinsically resistant. After incubation at 28°C for 48 h, each selected transconjugant was transferred to a microtiter plate well containing 100 μL of NB medium with kanamycin and erythromycin and incubated for 48 h at 28°C. Triplicates of each plate were generated by using a Steer replicator, a multiple inoculator composed of 96 specifically spaced inoculating rods corresponding in position to the wells of a microtiter plate, and after incubation, glycerol was added to each well at a final proportion of 30%. Plates were kept at -80°C until use.

In order to determine the insertion pattern of the *Tn5* *lux-lac Km2* transposon, 28 randomly selected insertion mutants were analyzed by Southern blot after digestion of the genomic DNA with SphI, using the *km* gene as a probe (Supplementary Table S1). Probe labeling, hybridization, and development were performed with the DIG DNA labeling and detection kit from Roche, following the manufacturer’s instructions. After hybridization, high-stringency washes of the membrane was carried out to remove the *km* probe and plasmid insertion events were analyzed in the same membrane using as a probe the *bla* gene previously amplified by PCR from the pUT mini-*Tn5 lux-lac Km2* vector (**Table 1**). Transposon stability was assessed by repeated subculture of some mutants in non-selective TSB. After 3 days subculture, the numbers of cultivable cells on TSA with and without kanamycin were determined. The percentages of kanamycin-resistant bacteria were determined in relation to total cells.

### Selection and Identification of Promoters by Using β-Galactosidase Activity as a Marker

In order to identify clones harboring transcriptional fusions between the *lacZY* operon and *Y. ruckeri* genes induced at 18°C, the library of transconjugants was replicated twice onto plates containing EMB medium supplemented with kanamycin. One of these plates was incubated at 18°C and the other one at 28°C for 40 and 24 h, respectively. After that, both replicas were analyzed to select those colonies having higher β-galactosidase activity, indicated by the colony having a more intense color, at 18°C than at 28°C. Clones of interest were submitted to a second screening in the same conditions and then kept at -80°C until use.

To identify the *Tn5 lux-lac Km2* transposon insertion site in the selected clones, genomic DNA from each clone was digested with PstI, XbaI, or SphI and then ligated into the pUC19 plasmid previously digested with the corresponding enzyme and dephosphorylated. The resulting ligation mixture was electroporated into *E. coli* S17-1*λpir* and clones of interest were further selected on 2x TY medium with ampicillin and kanamycin. These clones carried a DNA fragment harboring the kanamycin resistance gene from the transposon followed by a DNA fragment of variable length from the DNA located at the 3′position of the transposon insertion site. These fragments were sequenced using the TDKm6 primer (Supplementary Table S1) present at the end of the kanamycin resistance gene sequence and analyzed by RAST and BlastX programs.

### Complementation and Phenotypic Characterization of *acrR* and *osmY* Mutants

Complementation of *acrR* and *osmY* mutants was carried out by using plasmid pGBM5 (**Table 1**). *acrR*, *osmY*, and *osmY* and the adjacent gene *ytjA* (osmY^++^) were PCR amplified using the primers acrR-F/acrR-R, osmy-F/osmy-R and osmY-F/osmY-R2, respectively (Supplementary Table S1). All primers contained BamHI and PstI site at one end in order to clone the PCR-generated fragment into the BamHI-PstI restriction sites of the pGBM5 plasmid. The ligation mixture was introduced into *E. coli* DH5α *λpir* and transformants were selected on 2x TY medium with streptomycin. Restriction enzyme analysis, as well as PCR amplification of the generated plasmids, confirmed the correct structure and presence of the genes of interest. Constructions were transferred by electroporation to the respective mutants, and the appropriate clones were selected on TSA medium with kanamycin and streptomycin.

Susceptibility tests to antimicrobial agents and detergents were carried out in microtiter plates using twofold serial dilutions. Triplicate wells containing 5 × 10^4^ cells of each strain and the respective compound to be tested were incubated at 18°C for 24 h. The MIC was determined for each compound. The experiments were carried out in triplicate. The compounds assayed and the ranges of concentration were: acriflavine and chloramphenicol 0.02–40.96 μg/mL; ciprofloxacin 0.02–1.28 μg/mL; tetracycline 0.02–10.24 μg/mL; SDS and Triton X-100, 0.0015–25.6%.

To determine the effect of n-hexane and bile salts on bacterial growth 10 μL of a bacterial suspension of 10^8^ cfu/mL were deposited onto TSA medium in glass plates. After 10 min, the culture medium was covered with a 3 mm layer of n-hexane and plates were incubated at 18°C for 72 h. The effect of bile salts was assessed by spotting 10 μL of 10-fold serially diluted bacterial culture onto TSA media supplemented with 4% (w/v) bile salts in a proportion of 85:15 sodium cholate and sodium deoxycholate that, according to Denton et al. (1974), corresponds with that found in the rainbow trout gut.

For motility assays, 2 μl of overnight cultures of each strain were spotted onto TSA medium containing lactose 0.5% and 0.3% or 0.6% of agar; and in NA with 0.6% agar and different concentrations of glucose and lactose (glucose 0.25%, glucose 0.5%, lactose 0.75%, and lactose 0.5% + glucose 0.25%). Plates were incubated at 18 and 28°C for 3 days.

### Real-Time PCR Validation

Total RNA was extracted from two biological replicates of *Y. ruckeri* 150 grown at the early stationary phase (OD_600_ ≈ 1.1) in TSB at 28°C and 18°C. The cultures were fixed using RNA^TM^ Protect Bacterial Reagent (Qiagen Inc.) in a ratio of 1 mL of reagent per 0.5 mL of bacterial culture. Centrifugation was used to pellet the cells and RNA was extracted in RNase-/DNase-free water using the High Pure RNA Isolation Kit (Roche). After three treatments with RNase-free DNase (Ambion) to eliminate DNA contamination, total RNA was quantified using micro-spectrophotometry (Nanodrop ND-1000, Nanodrop Technologies, EEUU). The RNA quality was estimated using an Agilent 2100 Bioanalyzer and RNA samples with an RNA Integrity Number (RIN) above 9.5 were selected.

cDNA synthesis was performed from 1 μg of total RNA using the High Capacity cDNA Reverse Transcription Kit (Applied Biosystems, United States) and random primers according to the manufacturer’s instructions. Samples in which reverse transcriptase had not been added were used as negative controls. The cDNAs were subsequently quantified by real-time PCR amplification on an ABI PRISM 7900 Sequence Detection System (Applied Biosystems) with primers specifics to the *yrp1* (Yrp1-qF: 5′-TGCGCAAACCAATATCAGCG-3′; Yrp1-qR: 5′-TGCGCAAACCAATATCAGCG-3′), *acrR* (AcrR-qF: 5′-CGTGCTTATATCACCGGCCT-3′; AcrR-qR: 5′-AGGCATTGCGCGATCATTTC-3′), *osmY* (Osmy-qF: 5′-CGGTTAGCGAATATGCCGGT-3′; OsmY-qR: 5′-ACAAAACCACGCCATCGGTA-3′) and 16S (16S-F: 5′-TTTGTTGCCAGCACGTAATGGT-3′; and 16S-R: 5′-GCGAGTTCGCTTCACTTTGTATCT-3′) genes and using the SYBR Green PCR Master Mix (Applied Biosystems, United States).

Expression level results were standardized relative to the transcription level of the housekeeping gene 16S rRNA for each isolate. The relative change in the *yrp1*, *acrR*, and *osmY* was calculated as the ratio of reference target using the ΔΔCt, where Ct is the cycle threshold. Real-time PCR was carried out on two independent biological replicates each containing three technical replicates. The results are represented as the mean ± SD.

### Virulence Determination

For rapid virulence screening of mutant strains in the genes coding for AcrR, OsmY, hypothetical protein OEU24935.1, polymyxin resistance ArnC, hypothetical protein OEU21186.1, esterase YqiA and UDP-*N*-acetylglucosamine 4,6-deshydratase, a total of 10 fish, average weight 6–8 g, were intraperitoneally injected with 0.1 mL of 10^3^ cfu/mL of each mutant.

For virulence determination of *acrR^-^* and *osmY-*strains, 15 rainbow trout fry distributed in three groups were intraperitoneally injected with 0.1 mL of 10^7^ cfu/mL of each strain. The fish were kept in 70 L tanks at 18°C and mortalities were recorded each day for a week and compared with those produced by the parental strain. The data were analyzed with Chi-square test. *P* < 0.05 was considered statistically significant.

LD_50_ experiments by intraperitoneal injection with the parental, *acrR* mutant and *acrR^+^* complemented strains were carried out as described by Fernández et al. (2002). The doses injected ranged from 10 to 10^8^ cfu per fish and mortalities were followed up to 7 days. The LD_50_ values were calculated by the method of Reed and Muench (1938). Bath infection was carried out by immersion of the fish for 1 h in 10 L dechlorinated water containing a final concentration of 10^7^ cfu/mL of the different strains. The fish were then transferred to 70 L tanks and mortalities were followed up to 7 days. In both challenges, dead fish were withdrawn every day and samples from internal organs were spread onto TSA medium. After 48 h of incubation at 28°C several colonies of the predominant bacteria were identified by PCR in order to confirm that they corresponded to the previously injected strain.

All the experiments carried out with fish were authorized and supervised by the Ethics Committee of Oviedo University.

### Real-Time Visualization of *Y. ruckeri* Promoter Expression

Three groups of 30 fish weighing from 8 to 10 g were infected with *Y. ruckeri* *acrR^-^* and *osmY^++^* strains. The *acrR^-^* strain was used in both intraperitoneal and immersion infection experiments. Thus, one group of 30 fish was infected by intraperitoneal injection using 10^6^ cfu per fish and another group of 30 individuals was kept in a 10 L tank of dechlorinated water in contact with the *acrR* mutant at a concentration of 10^7^ cfu/mL for 1 h. Infection challenge using *osmY^++^* was only carried out by intraperitoneal injection following the procedure used for *acrR*. After infection, fish were transferred to tanks containing 70 L of dechlorinated water at 18°C. At 24 h intervals over a 7-day period, fish were euthanized by overdose of ethylene glycol monophenyl ether, dissected and analyzed with IVIS Imaging System (Xenogen) to monitor the bacterial progression and expression of *acrR* and *osmY* genes in the fish organs. Each experiment was repeated twice to ensure data accuracy.

To analyze gene expression using different rainbow trout tissues, each strain was spread on plates containing TSA medium for confluent growth. Then, fish tissues were deposited onto the medium. Plates were incubated at 18°C overnight and luminescence analyzed by Ivis Lumina.

## Results

### Development of a Vector to Generate a Dual Reporter *lux-lac* Transcriptional Fusion Library in *Y. ruckeri*

**FIGURE 1:**
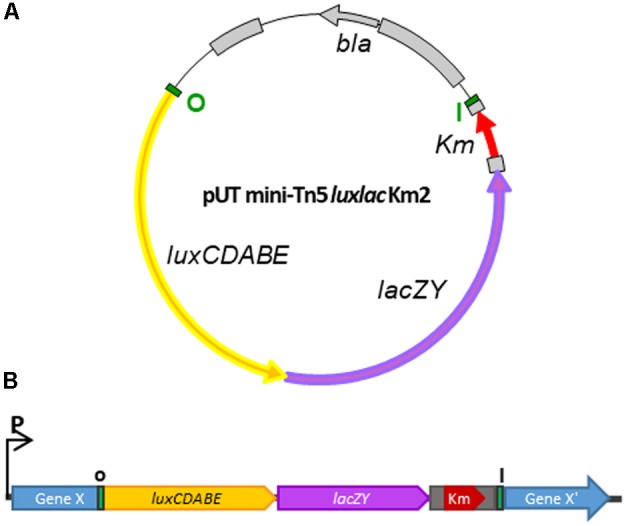
Schematic representation of the generation of a library of *Y. ruckeri* mutants by transposition. **(A)** Plasmid pUT mini-*Tn5 lux-lac Km2* carrying the modified mini-*Tn5* transposon harboring the *luxCDABE* and *lacZY* operons without promoters. **(B)** Transcriptional fusion between a putative *Y. ruckeri* chromosomal promoter and the *lux-lac* fusion operons generated after mini-*Tn5 lux-lac* insertion. *Bla*, resistance ampicillin; *Km*, resistance kanamycin; I-O terminal inverted *Tn5* sequences.

To create an appropriate dual reporter cassette for the analysis of differential gene expression in *Y. ruckeri* and also in other Gram-negative bacteria, we have modified the original pUT mini-*T5 Km2* plasmid, a *λpir*-dependent delivery vector. Inside the mini-*Tn5 Km2* transposon and at the 5′end of the kanamycin gene, the promoterless *luxCDABE* operon from *Photorhabdus luminescens* (Meighen and Szittner, 1992) and *lacZY* genes from the pIVET8 plasmid (Mahan et al., 1995) were inserted (**Figure 1A**). The resulting plasmid, named pUT-mini *Tn5 lux-lac Km2*, was transferred by conjugation from *E. coli* S17*λpir* to *Y. ruckeri* 150. Since the latter lacks the Pir protein necessary for the replication of the pUT plasmid, transconjugants must have randomly integrated the transposon (*Tn5 lux-lac Km2*) into their genomes. Insertion of the mini *Tn5* *lux-lac* *Km2* transposon into the *Y. ruckeri* genome generated, in many cases, transcriptional fusions between the promoter of the interrupted gene by the transposon and the *lux-lac* cassette (**Figure 1B**). In that way, the capture of promoters transcribing the *lux-lac* fusions was useful to determine *in vitro* and *in vivo* promoter regulation of different genes under specific conditions.

A library of 14,724 individual *Y. ruckeri* transconjugants harboring *Tn5 lux-lac Km2* insertions was generated. To determine if the transposon insertion in the genome of each cell was a random, plasmid-independent and unique event as it was previously described (Herrero et al., 1990), 28 kanamycin-resistant colonies were arbitrarily selected and subjected to Southern blot analysis. Each of the clones analyzed showed a single hybridizing fragment of a particular size when the kanamycin gene was used as a probe (Supplementary Figure S1A) indicating that the transposon insertion occurred randomly and only one time in the *Y. ruckeri* genome. When the ampicillin gene present in the plasmid was used as a probe not a single transconjugant showed a hybridizing signal (Supplementary Figure S1B). These results are consistent with those found by other authors.

### Characterization and Identification of Temperature-Regulated Promoters by Using *lux-lac* Reporters

**FIGURE 2:**
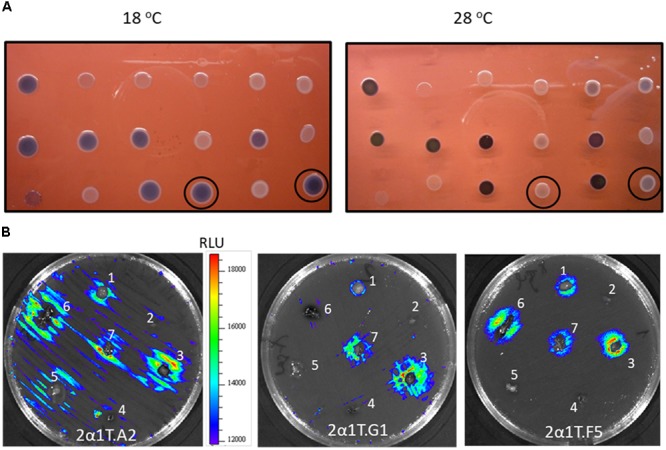
Temperature differential gene expression of *Y. ruckeri* clones containing the *Tn5-lux-lac* transposon insertion. **(A)** β-galactosidase activity on EMB medium after incubation at 18°C and 28°C. Circled colonies represent two examples of differential expression depending on the temperature. **(B)** Bioluminescence emission by different *Y. ruckeri* clones after 24 h of incubation at 18°C in the presence of different rainbow trout (*O. mykiss*) organs which were distributed on the surface of TSA plates: brain (1); heart (2); liver (3); spleen (4); adipose tissue (5); gut (6); gills (7), 2B. Bar indicating the correlation between luminescence (RLU) and color.

*Y. ruckeri* transconjugants were screened at 18° and 28°C for differential β-galactosidase activity in EMB medium (**Figure 2A**). A total of 168 clones (1.14%) displaying higher β-galactosidase activity, indicated by a more intense purple-black color at 18°C than at 28°C in the experimental conditions, were selected. Analysis of these clones by an Ivis Lumina apparatus showed that there was correspondence between the light emission level and β-galactosidase activity in most cases (data not shown).

The transposon insertion sites were mapped for 33 out of 168 selected clones and the identified genes are shown in **Table 2**. The growth culture patterns of several temperature-regulated mutants were similar to each other and also to the parental strain, indicating that the energy cost resulting from the differential expression of the *lux-lac* operons, both present in a single copy in the chromosome, had no major effect on the bacterial physiology (data not shown). For two clones, the existence of a linear correlation between cell number, in the range of 10^2^ and 10^7^ cfu, and luminescence with *R*^2^ values of 0.9593 and 0.9865, respectively, was also determined.

**Table 2:** Products of *Y. ruckeri* interrupted genes by the *Tn5-lux-lac* transposon.

Gene product	Accession number	Gene product	Accession number	Gene product	Accession number
Metalloprotease Yrp1	CAC39217.1	Galactose/methyl galactoside import ATP-binding protein	OEU26755.1	Tn7-like transposition protein	OEU24391.1
Diguanylate cyclase	OEU26487.1	Glycogen phosphorylase	OEU26568.1	ADP-ribosyltransferase exoenzyme family protein	KGA50131.1
Glycosyltransferase	OEU24749.1	4-alpha-glucanotransferase	OEU26314.1	PsiF repeat protein	EEQ00620.1
SAM-dependent methyltransferase	OEU24750.1	UDP-*N*-acetylglucosamine 4,6-dehydratase	OEU25693.1	Putative esterase (YqiA)	AJI94468.1
Serine/threonine protein kinase (anti-sigma regulatory factor)	OEU26573.1	D-Glycero-D-manno-heptose 1-phosphate guanosyltransferase	OEU25698.1	Hypothetical protein	OEU24382.1
Response regulator (YsrR)	OEU25024.1	O-antigen polymerase	ABY48117.1	Hypothetical protein	OEU24387.1
DNA-binding transcriptional repressor AcrR	OEU24368.1	O-acyltransferase	OEU26581.1	Hypothetical protein	KGA49879.1
Osmotically inducible protein OsmY	OEU26500.1	Putative peptidoglycan deacetylase	OEU25943.1	Hypothetical protein	OEU24935.1
Co/Mg/Ni transporter	OEU26742.1	Antitoxin of toxin-antitoxin stability system	OEU25145.1	Hypothetical protein	EEP98349.1
MFS transporter	OEU26751.1	Cell division protein (DamX)	EEP98349.1	Hypothetical protein	OEU25186.1
Phosphoporin PhoE	OEU26976.1	Polymyxin resistance protein ArnC	OEU24187.1	Hypothetical protein	OEU24718.1

In order to determine whether or not luminescence signals were useful in analyzing *Y. ruckeri* gene expression *ex vivo*, different randomly selected mutants were confluent spread on TSA medium. Tissue sections of several rainbow trout organs were deposited onto the culture and after 24 h of incubation at 18°C differences in the light emission displayed by the bacteria surrounding the different tissues were observed (**Figure 2B**). Thus, the gene interrupted in the 2a1T.A2 mutant showed highest light emission in liver, gills and gut tissues, whereas 2a1T.G1 and 2a1T.F5 mutants displayed the highest light emission in gills and liver, and gut and liver, respectively (**Figure 2B**).

Virulence determination was assessed in several of the selected mutants. Using intraperitoneal injection, 7 days post infection the parental strain produced 70% mortality, whereas mortalities for the selected mutants were: AcrR regulator, 80%; hyperosmotic protein OsmY, 0%; Hypothetical protein OEU24935.1, 30%; polymyxin resistance ArnC, 50%; Hypothetical protein OEU21186.1, 60%; Esterase YqiA, 50%; and UDP-*N*-acetylglucosamine 4,6-deshydratase, 0%.

### Analysis of the Mutations in the Temperature-Regulated Genes *acrR* and *osmY*

In order to check if the strategy developed to identify temperature-regulated genes allowed us to select the appropriate clones, two of the mutants harboring transcriptional fusions between the *acrR* and *osmY* promoters and the *lux-lac* operons were chosen for further analysis. The *acrR* mutant was selected because it was found to be as virulent as the parental strain, whereas the *osmY* mutant was attenuated. In this way, each of them was useful for the *in vivo* monitoring of the infection process: the *acrR* mutant as a positive control and the *osmY* mutant to confirm its attenuation in virulence which should revert with the introduction of *osmY* gene *in trans*.

First, the differences in β-galactosidase activity displayed by the two mutants in EMB at 18°C in relation to 28°C were quantified by the Miller method using *o*-nitrophenyl-β-D-galactopyranoside (ONPG) as a substrate. The results confirmed that both genes, *acrR* and *osmY*, were highly expressed at 18°C (2860.6 ± 158.3 and 786.6 ± 42.1 Miller units, respectively) in relation to 28°C (1439.8 ± 11.9 and 583.4 ± 15.4 Miller units). Moreover, in order to confirm the upregulation of *acrR* and *osmY* genes at 18°C compared to 28°C (used as a reference), SYBR Green qPCR was carried out using *yrp1* as positive control of expression at 18°C. According to the qRT-PCR analysis, *yrp1*, *acrR* and *osmY* transcription increased about three times at 18°C compared to 28°C (Supplementary Figure S2).

Once the higher expression of *acrR* and *osmY* genes at low temperature had been corroborated, their implication in the physiology and virulence of *Y. ruckeri* was investigated.

### Phenotypic Characterization and Virulence Determination of the *acrR* Mutant and Complemented Strain *acrR^+^*

The *acrR* gene from *Y. ruckeri* encodes for a 216 amino acid protein belonging to the TetR transcriptional repressor family, with high identity (80%) with the AcrR protein from *Y. pestis* (EFA48822.1) among other bacteria. This protein is a repressor which modulates the expression of the *acrAB* operon, which is located upstream and in the opposite direction of the *acrR* gene in the *Y. ruckeri* genome (Supplementary Figure S3). Binding of AcrR to the operator region located between the *acrR* and *acrAB* genes (Supplementary Figure S3) represses, in *E. coli*, both the expression of the *acrAB* operon, thus preventing its excessive expression, and also its own expression (Deng et al., 2013).

**FIGURE 3:**
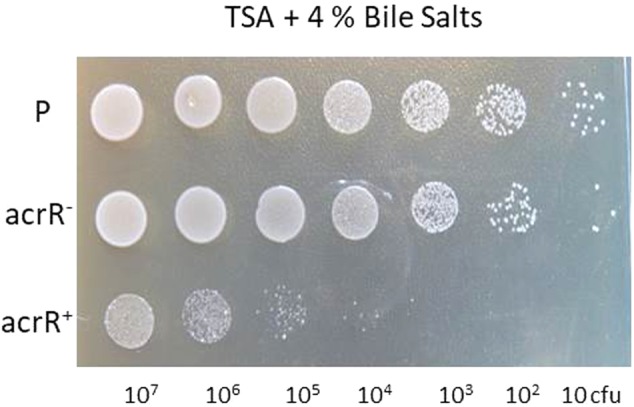
Involvement of the AcrR regulator in resistance to bile salts in *Y. ruckeri*. Ten microliters of 10-fold (diluted series) dilutions of *Y. ruckeri* strain cultures (corresponding to a range from 10^7^ to 10 cfu) were spotted onto TSA medium containing 4% bile salts and incubated at 18°C for 72 h. Parental (P), *acrR* mutant (*acrR^-^*), and complemented *acrR^-^* (*acrR^+^*) strains.

The AcrAB is part of the AcrAB-TolC pump involved in the expulsion of different toxic compounds from bacteria. Thus, as expected, the *acrR* mutant strain was more resistant than the parental strain to the antibiotics ciprofloxacin, chloramphenicol, tetracycline and also to acriflavine (**Table 3**). In the same way, the mutant strain turned out to be highly resistant to hexane, whereas the parental strain was sensitive (data not shown). The complemented mutant *acrR^+^* presented a similar phenotype to the parental strain, although it was a little more sensitive to the effect of all the antibiotics (**Table 3**). However, no differences were found between the parental and mutant strains in the sensitivity to detergents such as SDS and Triton X100. Interestingly, both strains were found to be resistant to bile salts when they were added at 4% to TSA medium, whereas the *acrR^+^* strain was highly sensitive (**Figure 3**).

**Table 3:** Effect of different compounds over *Y. ruckeri* *acrR* derivate strains.

Compound	*P*	*acrR^-^*	*acrR^+^*
Acriflavine	10,24	20,48	10,24
Ciprofloxacin	0,08	0,32	0,04
Chloramphenicol	5,12	10,24	2,56
Tetracycline	0,32	0,64	0,16
Triton X-100	12,8	12,8	ND
SDS	12,8	12,8	ND

**FIGURE 4:**
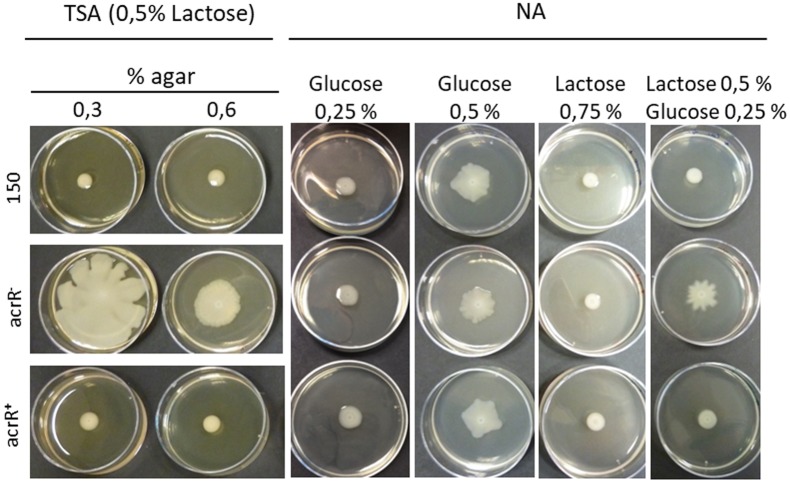
Effect of sugars on the motility of *Y. ruckeri*. Parental, *acrR^-^* and *acrR^+^* strains were spotted onto TSA media containing lactose 0.5% and 0.3% or 0.6% of agar; and in NA with 0.6% agar and different sugar percentages. Note that all the strains display motility when 0.5% glucose is present in the medium.

The *acrR^-^* mutant developed bigger colonies that the parental and *acrR^+^* complemented strains when they were grown in TSA medium containing lactose 0.5% (**Figure 4**). The reason is that in this medium, when the percentage of agar was lowered to 0.3% or 0.6%, the *acrR* mutant showed swimming and swarming motility, respectively. However, no motility at all was observed for the parental and complemented *acrR^+^* strains (**Figure 4**). Since TSA medium contains 0.25% glucose, a medium free of sugar such as NA was used to define the role of different carbohydrates in *acrR^-^* motility. Thus, when 0.25% glucose was added to this medium, no motility was observed in any strain, just as occurred in TSA medium (data not shown). However, when the percentage of glucose in NA medium was increased to 0.5%, all the strains showed motility (**Figure 4**). Other sugars such as galactose, lactose and arabinose did not induced motility in any strain when added to NA medium at 0.5% or 0.75% (data not shown). Nevertheless, the presence in the media of glucose at 0.25% and lactose at 0.5% only resulted in the motility of the *acrR^-^* strain, as occurred when the TSA supplemented with 0.5% lactose was used (**Figure 4**). All of these results can be explained by the β-galactosidase activity displayed by the *acrR* mutant that hydrolyses the lactose of the culture medium to glucose and galactose. Neither the parental strain nor the *acrR^+^* strain can use lactose, the first because of the lack of *lacZY* genes in the genome and the second as a consequence of the transcriptional repression of the *acrR* promoter, exercised by the overexpression of AcrR encoding in the plasmid used for complementation.

No clear differences in mortality were detected when parental and *acrR* mutant strains were administered (10^6^ cfu/fish) by intraperitoneal injection in three groups of five fish (data not shown). For this reason LD_50_ experiments were carried out. The results obtained confirmed that there were no significant differences between the parental (LD_50_ 6.3 × 10^2^ cfu) and mutant strains (LD_50_ 3.8 × 10^2^ cfu). Similarly, when the infection process was carried out by immersion, 90% of fish died 8 days post infection regardless of the strain inoculated. These results clearly indicate that the inactivation of the *acrR* gene in *Y. ruckeri* has no effect on virulence.

### Phenotypic Characterization and Virulence Determination of the *osmY* Mutant and Complemented Strains *osmY^+^* and *osmY^++^*

One of the selected clones presented the transposon inserted into an ORF encoding a 204 amino acid protein homolog to the OsmY protein of *E. coli*. The *Y*. *ruckeri* OsmY protein, according to Blast(p) program, has two BON conserved domains related to osmotic shock cell protection (pfam04972) (Yeats and Bateman, 2003; Zheng et al., 2015). *osmY* is flanked at the 5′end by the factor 3 encoding gene, involved in the release of the peptide chain, and at the opposite end by a gene encoding a 53 amino acid peptide containing the YtjA domain typical of many bacterial membrane proteins (NCBI accession number, COG5487). This genetic structure also appears in other *Yersinia* species as well as in *E. coli* K12, *Salmonella* Typhimurium and *Shigella flexneri*.

Growth curves in TSB medium of the *osmY* mutant and complemented *osmY^+^* and *osmY^++^* (*osmY*+*ytjA*) strains were similar to that of the parental strain (data not shown). Interestingly, when NaCl (1.5%) was added to TSB medium, up-regulation of the *osmY* gene was observed via bioluminescence emission, showing the relation between this gene and osmotic stress (Supplementary Figure S4).

As was inferred in previous experiments described above, the *osmY* mutant was found to be attenuated in virulence. Thus, injection experiments carried out with 10^6^ cfu per fish of parental and *osmY* mutant resulted in 93.3% mortality and 16.6% (*p*-value 0.0165), respectively, at 7 days post infection. When *osmY^+^* and *osmY^++^* strains were injected, partial virulence recovery reaching 40% (*p*-value 0.0183) fish mortality was observed, but only for the latter.

### *In Vivo* and *ex Vivo* Analysis of *acrR* and *osmY* Promoters

**FIGURE 5:**
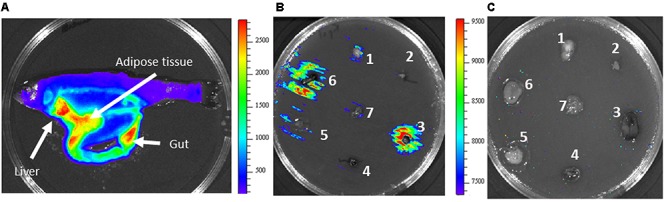
*In vivo* and *in vitro* bioluminescence emission by *Y. ruckeri* *acrR^-^* and *acrR^+^* strains. **(A)** Fish imaged at 4 days post infection carried out by intraperitoneal injection with the *acrR^-^* strain. Bioluminescence imaging of *acrR^-^* **(B)** and *acrR^+^* **(C)** strains in presence of different fish tissues deposited on TSA plates previously inoculated with the corresponding strain. Brain (1), heart (2), liver (3), spleen (4), adipose tissue (5), gut (6), gills (7). Color bar shows RLU level.

Transcriptional fusion between the *acrR* promoter and the *lux* operon was used for *in vivo* analysis of the infection process. Fish were infected both by injection or immersion and the light emitted by bacteria through fish tissues was monitored every 24 h by using the Ivis-Lumina system. Both models of infection began to produce luminescence 48 h post infection. At that time, high light intensity was located mainly in the liver, in the gut and in the adipose tissue (**Figure 5A**). A similar result was found when the *acrR* mutant was confluent grown on TSA plates containing different trout tissues. As can be observed in **Figure 5B**, maximum luminescence emitted by the *acrR* mutant corresponded to liver, just as was seen *in vivo,* although light was also detected in the gut. Interestingly, no luminescence was detected in the adipose tissue. *Yersinia ruckeri acrR^+^* did not showed bioluminescence in presence of any fish tissue (**Figure 5C**).

**FIGURE 6:**
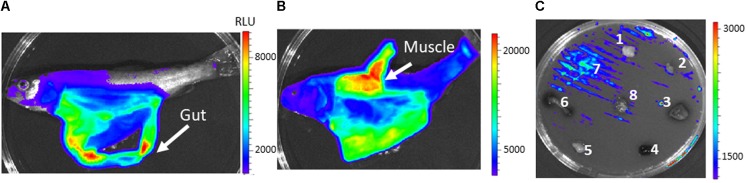
*In vivo* and *in vitro* bioluminescence emission by *Y. ruckeri* *osmY^++^* strain. Fish intraperitoneally infected with the *osmY^++^* strain were visualized at 3 days **(A)** and 4 days **(B)** post infection. *In vitro* expression of *osmY* in response to different fish tissues deposited on TSA plates **(C)**. Brain (1), heart (2), liver (3), spleen (4), adipose tissue (5), gut (6), muscle (7), gills (8). Color bar shows RLU level.

As expected, according to the virulence attenuation, at LD_50_ doses no light emission was detected at any time post infection when fish were intraperitoneally injected with the *osmY* mutant and *osmY^+^* strains and further examined under Ivis-Lumina equipment (data not shown). However, when the *osmY^++^* complemented strain was used, *osmY* expression was highly detected in the gut (**Figure 6A**), although in many cases high levels of expression in the muscle tissue were found at the end of the infection process, in dead fish (**Figure 6B**). A similar result was found when rainbow trout tissues were deposited onto TSA media, previously inoculated with *Y. ruckeri* *osmY^++^* strain for confluent growth. Thus, the highest luminescence intensity after 24 h of incubation was observed in bacteria surrounding the muscle (**Figure 6C**).

## Discussion

In this study, a novel mini *Tn5* transposon plasmid, pUT-mini *Tn5 lux-lac Km2*, was constructed on the backbone of pUT *Tn5 Km2* plasmid (de Lorenzo et al., 1990). The main advantage of mutagenesis with this transposon is that its insertion in the bacterial genome not only results in a knockout mutation but also may lead to the production of transcriptional fusions between the promoterless *luxCDABE* and *lacZY* operons and the promoter of the interrupted gene. This study demonstrated that the *Tn5 lux-lac Km2* transposon was integrated randomly into the genome of *Y. ruckeri* and was stable in the absence of selective pressure. Additionally, it was confirmed that expression of *lux-lac* reporter genes has no effect on the growth rate of *Y. ruckeri*.

A library of *Y. ruckeri* *Tn5 lux-lac Km2* mutants was screened, via detection of β-galactosidase activity on EMB medium, for differential expression depending on temperature. Thus, a total of 168 clones showed higher expression at 18°C, the temperature at which outbreaks occurred, than at the optimal growth temperature (28°C). In 33 of those clones the gene interrupted was identified (**Table 2**). Interestingly, one of the selected genes was the *yrp1*, encoding for the metalloprotease Yrp1 (CAC39217.1) (Fernández et al., 2002, 2003). This gene has already been reported to be regulated by temperature, its expression being more active at 18°C than at 28°C (Fernández et al., 2002). Additionally, up to three different genes disrupted by the transposon encode for proteins involved in the synthesis of legionaminic acid (OEU25698.1, OEU25693.1, ABY48117.1), which strongly supports the involvement of temperature in the regulation of this process (Welch and LaPatra, 2016; Cascales et al., 2017). All of this strongly suggests that the strategy aimed at selecting promoters whose activity is regulated by temperature (highly expressed at 18°C in relation to 28°C) was the appropriate one. The selected mutants were grouped according to specific functions:

One group of mutants presented transposon insertions in genes related to response to environmental changes. Among them, the *yfiN* gene encoding the diguanylate cyclase (OEU26487.1) involved in the synthesis of cyclic di-GMP, a bacterial second messenger related to the control of many bacterial cellular functions such as changes from the transition between sessile and motile bacterial states (Römbling et al., 2013) and virulence (Raterman et al., 2013; Xu et al., 2016). Two other mutants presented transposon insertion in a putative operon of two genes encoding for a glycosyltransferase (OEU24749.1) and a SAM-dependent methyltransferase (OEU24750.1). Both kinds of enzymes have been previously linked to virulence in bacteria such as *Haemophilus parasuis* (Zhou et al., 2016), *Mycobacterium tuberculosis* (Berney et al., 2015), and *Klebsiella pneumoniae* (Ye et al., 2016). Three transcriptional regulators were also found. One of them encodes for a serine/threonine kinase (OEU26573.1) homologous to the anti-sigma factor RsbW from *Y. frederiksenii* which is involved in stress response through modulation of the sigma B factor (Hughes and Mathee, 1998). Another was the regulator YsrR (OEU25024.1), a member of the two component regulatory system YsrRS that in *Y. enterocolitica* is involved in the regulation of the type III secretion system Ysa-YsP (Walker and Miller, 2004). The third was the AcrR regulator (OEU24368.1), which represses the AcrAB-TolC pump expulsion system (Deng et al., 2013) involved in resistance to toxic compounds. This gene as well as *osmY* (OEU26500.1), a gene induced under osmotic stress, were further analyzed in this work.

Molecular exchanges between bacteria and their environment is mediated by multiple and complex systems. Three genes were selected in relation to this process: *mgtE* (OEU26742.1) involved in the transport of magnesium, cobalt and nickel and the genes encoding for a MSF family transporter (OEU26751.1) and the PhoE phosphoporin (OEU26976.1). All of them have been previously linked to virulence (Osorio et al., 2004; Andersson and Hughes, 2010; Chen et al., 2013; Coffey et al., 2014).

Another group of interrupted genes has a relationship with transport and the metabolism of sugars. Such is the case of *mglA*, a component of an ABC transport system (OEU26755.1) involved in galactose uptake (Harayama et al., 1983); a gene coding a glycogen phosphorylase (OEU26568.1) and another one, *malQ* (OEU26314.1) which forms part of *malPQ* operon involved in the uptake and degradation of maltodextrin which is the preferential carbon source in *E. coli* (Boos and Shuman, 1998). In *V. cholera*, it has been described that *malQ* plays a role in the virulence of the bacterium (Lang et al., 1994).

A major group of selected genes had a relationship with the bacterial membrane and wall. Thus, the interrupted genes of three different clones form part of a cluster encoding for proteins involved in the synthesis of legionaminic acid, a component of the LPS structure. The encoded proteins correspond with a UDP-*N*-acetylglucosamine 4,6-dehydratase (OEU25693.1), a D-glycerol-D-mano-heptosa-1-phosphate guanosyltransferase (OEU25698.1) and an *O*-antigen polymerase (ABY48117.1). This cluster is only present in *Y. ruckeri* serotype O1 (Welch and LaPatra, 2016; Cascales et al., 2017), and vaccination with a strain carrying a deletion of the second gene of the cluster encoding for the *nab2* gene, a nonulosonic acid biosynthesis gene (*nab* gene), resulted in absence of protection against yersiniosis infection, showing the importance of this cluster for ERM bacterin vaccine efficiency (Welch and LaPatra, 2016). According to this and to data reported from other bacteria such as *Legionella pneumophila* (Lüneberg et al., 1998) and *Campylobacter jejuni* (Zebian et al., 2016), it is possible that legionaminic acid is involved in the virulence of *Y. ruckeri*. Additional selected genes related to the cellular wall were an *O*-acyltransferase and a polysaccharide deacetylase which are probably involved in peptidoglycan biosynthesis. Two other genes with higher expression at 18°C than at 28°C are those encoding an *O*-acyltransferase (OEU26581.1) and a putative deacetylase (OEU25943.1) probably involved in peptidoglycan synthesis. In *Streptococcus iniae* the polysaccharide deacetylase Pdi is involved in virulence in the hybrid striped bass model and is necessary for survival in whole fish blood.

Another identified gene was related to antitoxin StbD (OUE25145.1) from the StbD/E toxin-antitoxin system (TA). TA systems are related to multiple functions in bacteria and, recently, some authors have suggested that they could in some cases be directly or indirectly related to infection processes in bacteria (Lobato-Márquez et al., 2016).

A mutant in the *damX* gene coding for a protein involved in cell division (EEP98349.1) was also isolated. In *S. typhi* (Leclerc et al., 1998) and *E. coli* (Khandige et al., 2016) this gene was necessary for full virulence.

Other two selected mutants had interrupted the gene encoding the ArnC protein (OEU24187.1), which confers resistance to polymyxin, an antibiotic used in the therapy of multidrug-resistant Gram-negative bacterial infections (Evans et al., 1999), and a gene encoding a protein similar to the protein TnsC (OEU24391.1), which is part of the transposition machinery of the *Tn7* transposon. This kind of transposon is widespread in bacteria and facilitates the accumulation of mobile DNA within the *att*Tn*7* site in the chromosome leading to the formation of genomic islands (Parks and Peters, 2007).

PsiF (EEQ00620.1) is an inducible protein under phosphate starvation (Metcalf et al., 1990). Its expression is under the control of the PhoB-PhoR two-component system which is involved in virulence, motility and biofilm formation, among others (Lamarche et al., 2008). The selection at outbreak temperature of the gene coding PsiF in *Y. ruckeri* could be related to a response of the bacterium to phosphate limitation during the infection process.

An interesting selected gene is that encoding an ADP-ribosyltransferase exoenzyme (KGA50131.1). A large number of these enzymes are toxins involved in covalent modification of host proteins leading to a pathological manifestation. That is the case for different toxin produced by the genus *Clostridium* (Stiles et al., 2014), the SpvB virulence factor of *Salmonella enterica* (Lesnick et al., 2001) and A toxin of *Pseudomonas aeruginosa* (Wick et al., 1990), among others.

Some of the identified genes encode for hypothetical proteins. It is the case of the gene encoding for the hypothetical protein OEU2487.1 harboring a PDDEXK_7 domain characteristic of nucleases involved in DNA restriction methylation-dependent. The encoding gene forms part of an operon together with a gene coding for and AAA type ATPase involved in DNA repair. In the same way, the gene coding for the hypothetic protein KGA49879.1 seems to be part of an operon related to sugar metabolism. Additionally, the gene encoding for the hypothetical protein OEU24382.1 probably forms part of an operon with the gene encoding for a GTP pyrophosphokinase, homolog to RelA/SpoT, which is associated with virulence in different pathogenic bacteria (Klinkenberg et al., 2010; Nguyen et al., 2011; Zhu et al., 2016).

Finally, mention should be made of the gene encoding the esterase YqiA (AJI94468.1), which forms part of an operon including three other genes encoding for an ADP-ribose pyrophosphatase, a membrane protein and an cAMP phosphodiesterase involved in different cellular process, including the expression of virulence factors in *V. cholera* (Skorupski and Taylor, 1997) and *V. vulnificus* (Jeong et al., 2001; Choi et al., 2002).

### Role of *acrR* and *osmY* Genes and Their Expression During Infection

As expected, the absence of the *acrR* gene in *Y. ruckeri* resulted in an increase in the resistance to a wide variety of toxic compounds that are extruded by the AcrAB-TolC system, including chloramphenicol or tetracycline as well as n-hexane, among others. Epidemiological data indicate that in *Y. ruckeri,* tetracycline resistance genes also confer resistance to oxytetracycline, a compound used in the treatment of red mouth disease (Balta et al., 2010). According to the study of Balta et al. (2010) 47.6% of isolated oxytetracycline-resistant *Y. ruckeri* strains harbored the *tetA* and *tetB* genes. In the other strains, resistance to oxytetracycline could be related to an increase in the AcrAB-TolC system activity, resulting from a simple *acrR* mutation which would be further selected during antibiotic treatments.

Bile salts induce the expression of the *E. coli* AcrAB-TolC system (Rosenberg et al., 2003). Although the expression of this pump is subject to complex regulation, it is activated by compounds which come into contact with the bacteria. In this way, *Y. ruckeri*, as an intestinal pathogen, is highly resistant to bile salts given that 4% of these compounds allowed the growth of both parental and *acrR^-^* strains. Interestingly, the complemented *acrR* strain was more sensitive to bile salts as well as to tetracycline, ciprofloxacin and chloramphenicol than the parental and mutant strains. This indicates that in the complemented strain the AcrAB pump is highly repressed by AcrR, which in turn suggests that the AcrAB pump is involved in the expulsion of bile salts from *Y. ruckeri*.

It has been previously reported that in *E. coli*, AcrR is involved in swimming and swarming motilities, both mediated by flagella, and necessary to evade toxic compounds (Inoue et al., 2007; Kim et al., 2016). In this sense, it seems that the role of AcrR goes beyond the repression of the AcrAB-TolC system. However, our data indicate that the increase in motility detected for the *Y. ruckeri* *acrR* mutant strain was not a consequence of this mutation, but of the presence in the culture media of a minimum proportion of 0.5% of glucose, part of which originated from lactose degradation. Thus, in the presence of 0.5% glucose, the parental, *acrR* mutant and complemented strains displayed motility. In *Y. ruckeri*, this relationship between glucose and motility seems to be specific since other sugars did not produce any effect on motility. In *Salmonella* Typhimurium and *E. coli*, the presence of glucose in the medium was necessary for swarming, probably because it provides the energy needed for the process (Harshey, 1994; Inoue et al., 2007; Kim et al., 2016). However, gliding motility in *Clostridium perfringens* is repressed by sugars such as glucose, lactose and galactose (Mendez et al., 2008) and swarming motility in *Pseudomonas aeruginosa* is limited by the presence of glucose (Shrout et al., 2006), indicating that effect of glucose in the motility of bacteria is species-specific.

In spite of being a gene regulated by temperature, *acrR* was not involved in the infection process when *Y. ruckeri* was infected either by intraperitoneal via or by bath immersion. Different studies indicate that the interruption of the AcrAB-TolC system resulted in virulence attenuation of *S.* Typhimurium (Lacroix et al., 1996), *Klebsiella pneumoniae* (Padilla et al., 2010), *Enterobacter cloacae* (Perez et al., 2012), *Francisella tularensis* (Bina et al., 2008) and the fish pathogen *E. tarda* (Hou et al., 2009). However, there are some exceptions to this, such as the case of *Y. pestis* in which the AcrAB does not play an essential role in the pathogenic process (Lister et al., 2012), something that also seems to occur in *Y. ruckeri*.

Although its role is still unknown, induction of the *osmY* gene of *Y. ruckeri* in presence of NaCl confirms the relation between this gene and osmotic stress, as occurred in *E. coli* (Yim and Villarejo, 1992; Yim et al., 1994). The fact that mutation of the *osmY* gene resulted in a decrease in the virulence of the bacterium showed its importance in the development of the infection process. Perhaps the role it plays has to do with a response to the high molarities present in the fish gut. In any case, not only is the *osmY* gene involved in the stress response, but also the adjacent one, *ytjA*, given that complementation of the *osmY* mutant strain with both *osmY* and *ytjA* genes was necessary to achieve partial virulence recovery. There are not much data on the role and involvement of *osmY* in bacterial virulence but some indirect relationship was found in *S.* Typhimurium (Bader et al., 2003). Zheng et al. (2015) suggested that *osmY* could play a role during the initial period of infection where the bacterium has to survive osmotic gut stress. This gene was also linked to the infection processes caused by *Cronobacter sakazakii* (Ye et al., 2015) and *E. coli* (Dong and Schellhorn, 2009).

Using the *lux* operon as reporter gene, it was possible to follow the *acrR* and *osmY* gene expression in different fish tissues. Interestingly, *in vivo* experiments showed that light emission of *acrR* was higher in gut, liver and adipose tissue, where toxic compounds are usually accumulated, whereas *osmY* showed higher levels of light in the gut, probably mediated by the osmotic stress generated in the bacterium in this organ. Nevertheless, the high level of luminescence detected after septicaemia in muscle tissue in many of the fish analyzed indicates that *osmY* expression could depend on the infection state.

These results are in agreement with those obtained on plates using rainbow trout tissues. Therefore, maximum luminescence resulting from *acrR* promoter activity was also detected in gut and liver, although, adipose tissue did not give a high level of light probably because this tissue, once out of the fish, was rapidly altered by fatty acid oxidation. In the same way, the *osmY* gene showed the highest light emission in muscle tissue. Bioluminescence-based bacterial reporter genes are useful to identify and study *in vitro* and *in vivo* gene regulation (Méndez and Guijarro, 2013; van Zyl et al., 2015). This result, together with that obtained using randomly selected clones cultivated in the presence of rainbow trout tissues, indicates that plate assays seem to be useful to determine *Y. ruckeri* differential gene expression in trout tissues. This approach allows the number of fish used in the experiments to be reduced, thus making the analyses easier and faster.

## Author Contributions

DC performed most of the experiments and analysis of results. AG-T participated in some of the experiments and analysis of results. JM participated in experimental design, experimental work, and critically revised the results. JG participated in experimental design, data analysis, and wrote the paper. DC, JM, AG-T, and JG drafted the first version of the manuscript. All authors reviewed the final version of the manuscript.

## Conflict of Interest Statement

The authors declare that the research was conducted in the absence of any commercial or financial relationships that could be construed as a potential conflict of interest.
