# Supplementary material for: Temperature-Dependent Gene Expression in Yersinia ruckeri: Tracking Specific Genes by Bioluminescence During in Vivo Colonization
Source: Front Microbiol. 2018 May 25;9:1098. doi: 10.3389/fmicb.2018.01098 (PMC5981175; doi:10.3389/fmicb.2018.01098)
Supplement: Supplementary file 1 [file Table_1.DOCX]

**Table 1S**

| Genes | Primers |
| --- | --- |
| *km* | Km1: 5ˈ-CATATTCAACGGGAAAC-3ˈ |
|  | Km2: 5ˈ-ATCAGGATTATCAATACCA-3ˈ |
| *bla* | Bla1: 5'-GTGTCGCCCTTATTCCCTTT-3' |
|  | Bla2: 5'-GGCACCTATCTCAGCGATCT-3' |
| *acrR* | acrR-F:5'-TTTATCTGCAGTCCTATAAGTACCAAGCTG-3’ |
|  | acrR-R:5'-TTTAAGGATCCAATGCGTTAAATAATGACT-3' |
| *osmY* | osmY-F:5'-ATGCCTGCAGTCATCGGGGTATTTCATT-3' |
|  | osmY-R:5'-ATGCGGATCCCCAACGGCCAGAAGCAG-3' |
| *osmy-ytjA* | osmY-F:5'-ATGCCTGCAGTCATCGGGGTATTTCATT-3' |
|  | osmY-R2:5'-ATGCGGATCCCATAGTGATGTCACCCT-3' |

**Oligonucleotides used in this work.**
