# Supplementary figures and images for: Temperature-Dependent Gene Expression in Yersinia ruckeri: Tracking Specific Genes by Bioluminescence During in Vivo Colonization
Source: Front Microbiol. 2018 May 25;9:1098. doi: 10.3389/fmicb.2018.01098 (PMC5981175; doi:10.3389/fmicb.2018.01098)

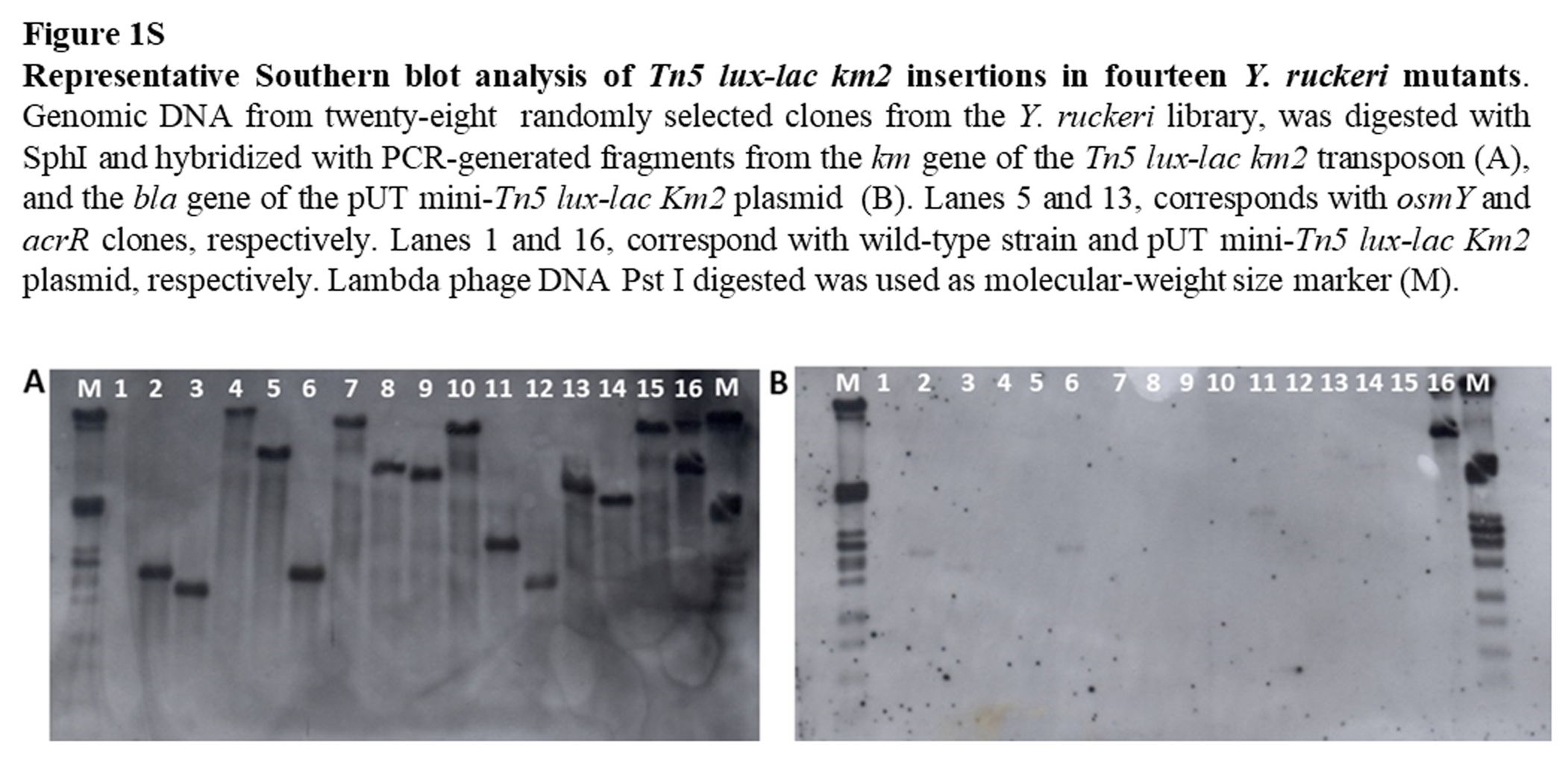

Supplement: Supplementary file 2 [file Image_1.PNG]

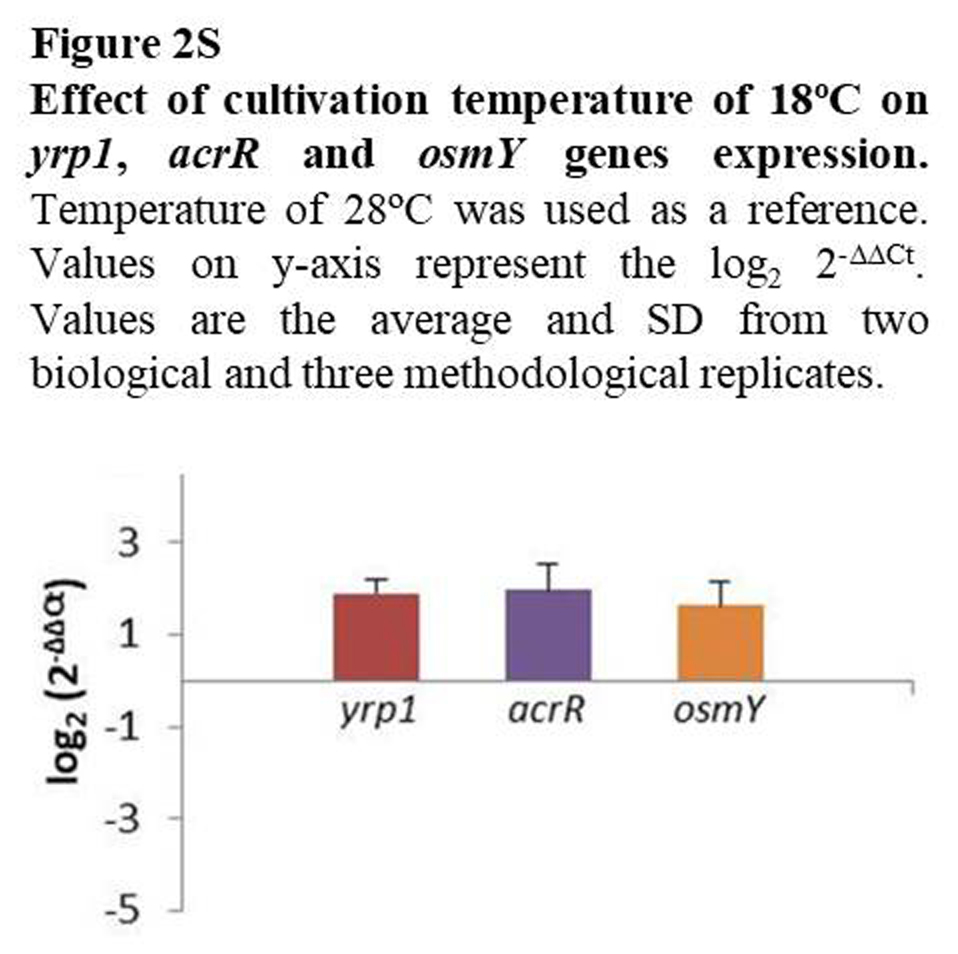

Supplement: Supplementary file 3 [file Image_2.JPEG]

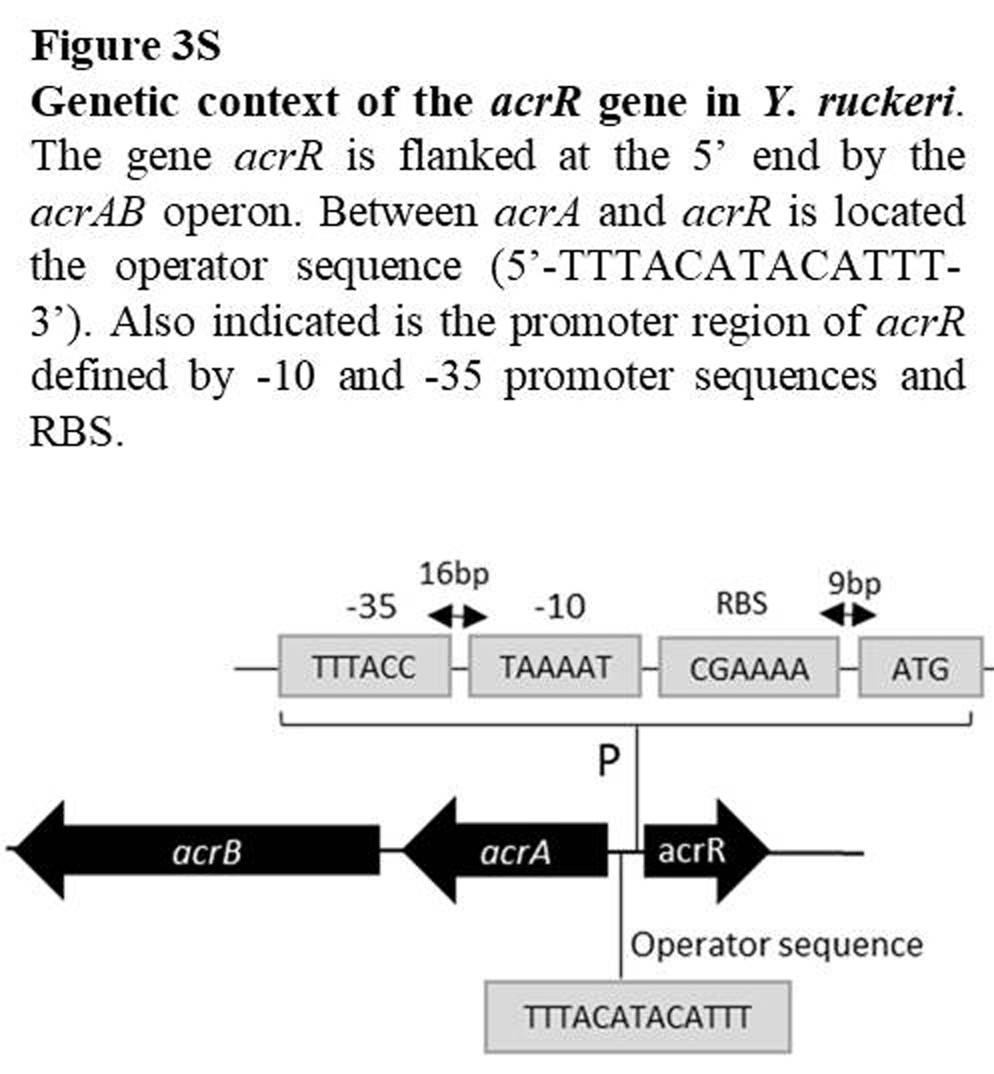

Supplement: Supplementary file 4 [file Image_3.JPEG]

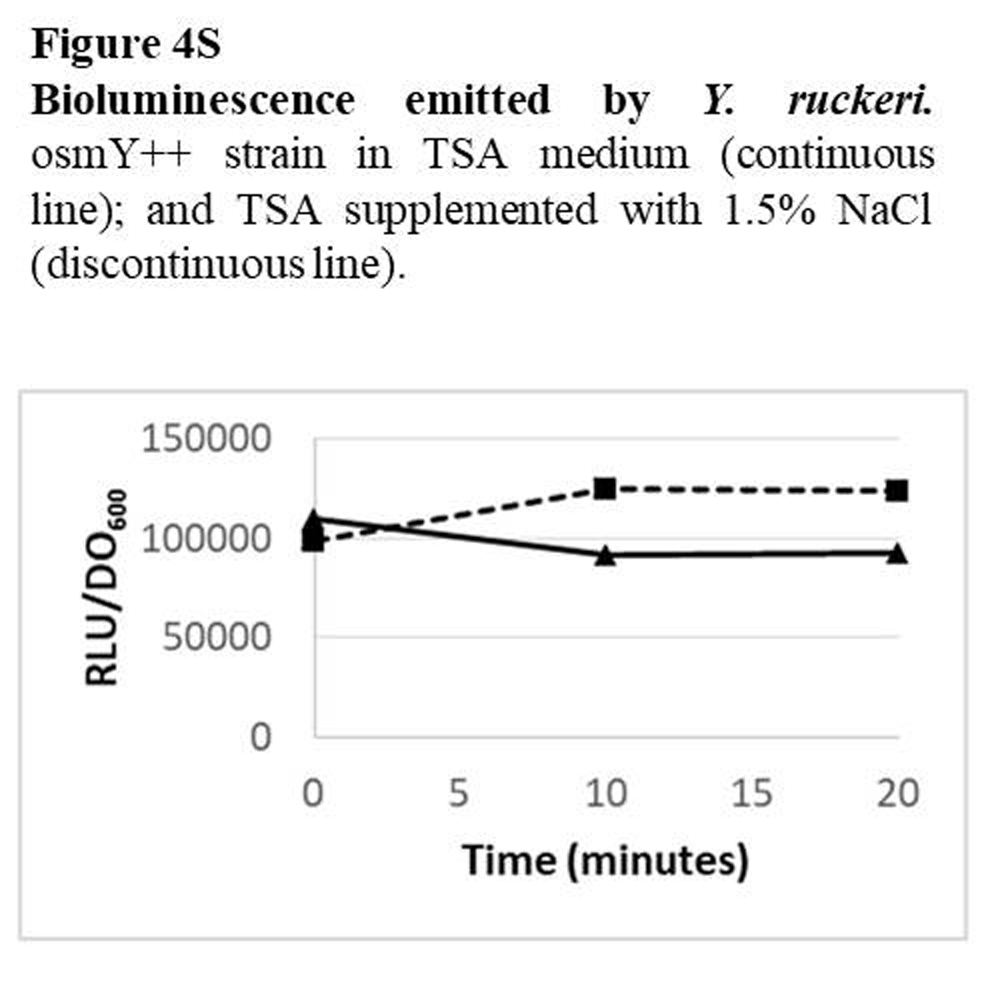

Supplement: Supplementary file 5 [file Image_4.JPEG]
